# Objective Burn Scar Assessment in Clinical Practice Using the Cutometer^©^: Introduction and Validation of a Standardized Measurement Protocol

**DOI:** 10.1093/jbcr/irac154

**Published:** 2022-10-27

**Authors:** Felix J Klimitz, Hubert Neubauer, Annette Stolle, Sabine Ripper, Simeon C Daeschler, Martin Aman, Arne Boecker, Benjamin Thomas, Ulrich Kneser, Leila Harhaus

**Affiliations:** Department of Hand, Plastic and Reconstructive Surgery, Burn Center, BG Trauma Center Ludwigshafen, Plastic- and Hand Surgery, University of Heidelberg, Ludwig-Guttmann-Str. 13, 67071, Ludwigshafen, Germany; Department of Hand, Plastic and Reconstructive Surgery, Burn Center, BG Trauma Center Ludwigshafen, Plastic- and Hand Surgery, University of Heidelberg, Ludwig-Guttmann-Str. 13, 67071, Ludwigshafen, Germany; Department of Hand, Plastic and Reconstructive Surgery, Burn Center, BG Trauma Center Ludwigshafen, Plastic- and Hand Surgery, University of Heidelberg, Ludwig-Guttmann-Str. 13, 67071, Ludwigshafen, Germany; Department of Hand, Plastic and Reconstructive Surgery, Burn Center, BG Trauma Center Ludwigshafen, Plastic- and Hand Surgery, University of Heidelberg, Ludwig-Guttmann-Str. 13, 67071, Ludwigshafen, Germany; Department of Hand, Plastic and Reconstructive Surgery, Burn Center, BG Trauma Center Ludwigshafen, Plastic- and Hand Surgery, University of Heidelberg, Ludwig-Guttmann-Str. 13, 67071, Ludwigshafen, Germany; Department of Hand, Plastic and Reconstructive Surgery, Burn Center, BG Trauma Center Ludwigshafen, Plastic- and Hand Surgery, University of Heidelberg, Ludwig-Guttmann-Str. 13, 67071, Ludwigshafen, Germany; Department of Hand, Plastic and Reconstructive Surgery, Burn Center, BG Trauma Center Ludwigshafen, Plastic- and Hand Surgery, University of Heidelberg, Ludwig-Guttmann-Str. 13, 67071, Ludwigshafen, Germany; Department of Hand, Plastic and Reconstructive Surgery, Burn Center, BG Trauma Center Ludwigshafen, Plastic- and Hand Surgery, University of Heidelberg, Ludwig-Guttmann-Str. 13, 67071, Ludwigshafen, Germany; Institute of Medical Biometry and Informatics (IMBI), University of Heidelberg, Im Neuenheimer Feld 130.3, 69120 Heidelberg, Germany; Department of Hand, Plastic and Reconstructive Surgery, Burn Center, BG Trauma Center Ludwigshafen, Plastic- and Hand Surgery, University of Heidelberg, Ludwig-Guttmann-Str. 13, 67071, Ludwigshafen, Germany; Department of Hand, Plastic and Reconstructive Surgery, Burn Center, BG Trauma Center Ludwigshafen, Plastic- and Hand Surgery, University of Heidelberg, Ludwig-Guttmann-Str. 13, 67071, Ludwigshafen, Germany

## Abstract

An objective burn scar assessment is essential to informed therapeutic decision-making and to monitor scar development over time. However, widely employed scar rating scales show poor inter-rater reliability. For this study we developed a standardized measurement protocol for the Cutometer^©^ applicable for objective burn scar assessment in everyday clinical practice. We developed a measurement protocol for the Cutometer^©^ MPA 580 including a scar site relocation technique based on anatomical landmarks. The protocol emerged through several steps: Identifying key factors for valid and reliable measurements, preliminary testing, specification of technical details, refining the protocol and final testing. Consecutively, the protocol was validated for inter-rater reliability by assessing 34 burn scars in 17 patients by four clinicians and computing an Intra-class Correlation Coefficient (ICC). Parameter *R*0, representing scar pliability, was identified as the best suited output parameter yielding excellent inter-rater reliability for average measures (ICC 0.92 [95% CI 0.86; 0.96]) and acceptable reliability for single measures (ICC: 0.74 [0.61; 0.84]). The pressure applied on the measuring probe was identified as an influential confounding factor for reliable measurements. Rater gender did not influence reliability of measurements. The introduced standardized measurement protocol for the Cutometer^©^ MPA 580 enables an objective and reliable burn scar assessment for clinical as well as research purposes.

Severe burn injuries often result in hypertrophic scars leading to lifelong disability and burdensome sequelae.^1^ To alleviate or even prevent hypertrophic scarring following burn injury, a number of surgical and non-surgical treatment options are available.^2^ However, an adequate therapy of burn scars depends on a reliable scar evaluation in order to identify indications and track the treatment response. Presently, the clinical assessment of burn scars still relies on subjective, error-prone scar rating scales hampering a confident clinical decision-making.^3^ Although those scar rating scales, including the Vancouver Scar Scale (VSS) and Patient and Observer Scar Assessment Scale (POSAS), are considered cost-effective and easy to use,^4–7^ it has been demonstrated that their accuracy depends on the examiner’s experience. As a consequence, their reliability among individual clinical raters is often poor^8–10^ This may hinder a timely treatment introduction and thereby limit the efficacy of modern therapies, particularly in out-patient care with infrequent patient encounters.

Technology-based scar assessment tools, increasingly used in burn scar research, may address this issue by providing an objective assessment of scar tissue characteristics.^6,11–13^ This may help to identify scars prone to hypertrophy early on and thereby potentially facilitate a timely and targeted introduction of therapeutic and rehabilitative interventions. In an effort to introduce comprehensive burn scar assessments for clinical routine, Lee et al^14^ recently suggested a combined panel of several objective scar assessment tools in combination with subjective scales to assess multiple scar properties simultaneously. Although very informative, such time-consuming and elaborate measurement procedures restrict their applicability in clinical routine with often limited time and personnel. Thus, to this day, there is no consensus on how to balance clinical feasibility with reliability in order to obtain an objective burn scar assessment.

To address the issue of clinical practicability and cost-effectiveness, we focused on a single objective scar assessment tool. We chose the Cutometer^©^ MPA 580 (Courage & Khazaka Electronic GmbH, Cologne, Germany), a non-invasive suction device applied to quantitatively assess mechanical skin properties.^13,15^

In this study, we introduce and validate a method for a precise, yet time-efficient relocation protocol to provide high inter-rater reliability while ensuring clinical feasibility.

## METHODS

### Study Design

A search of the literature on available objective burn scar assessment tools was performed with particular regard for tools tested for validity and inter-rater reliability as well as outcome parameters for clinically relevant scar properties. Among the available tools, we identified the Cutometer^©^ MPA 580 as best suited as it is the most extensively used device in the literature^12^ and provides information on scar pliability an essential clinically relevant parameter for burn scar assessment. Developed and reliability-tested for healthy skin, the Cutometer^©^ has found broad application in the assessment of multiple types of scars.^9,10,16–18^ However, a key limiting factor for its application in clinical practice is the diverging use of various, often time-consuming procedures to relocate a measured scar site by following examiners. Consecutively, a standardized procedure for objective burn scar assessment applying the Cutometer^©^ in everyday clinical practice including a novel method of scar site relocation was developed. Afterwards, this protocol was validated for inter-rater reliability in a prospective, non-blinded single-arm observational study conducted at the burn center of our institution.

### Measurement Device: Cutometer^©^ MPA 580

**Figure 1.**
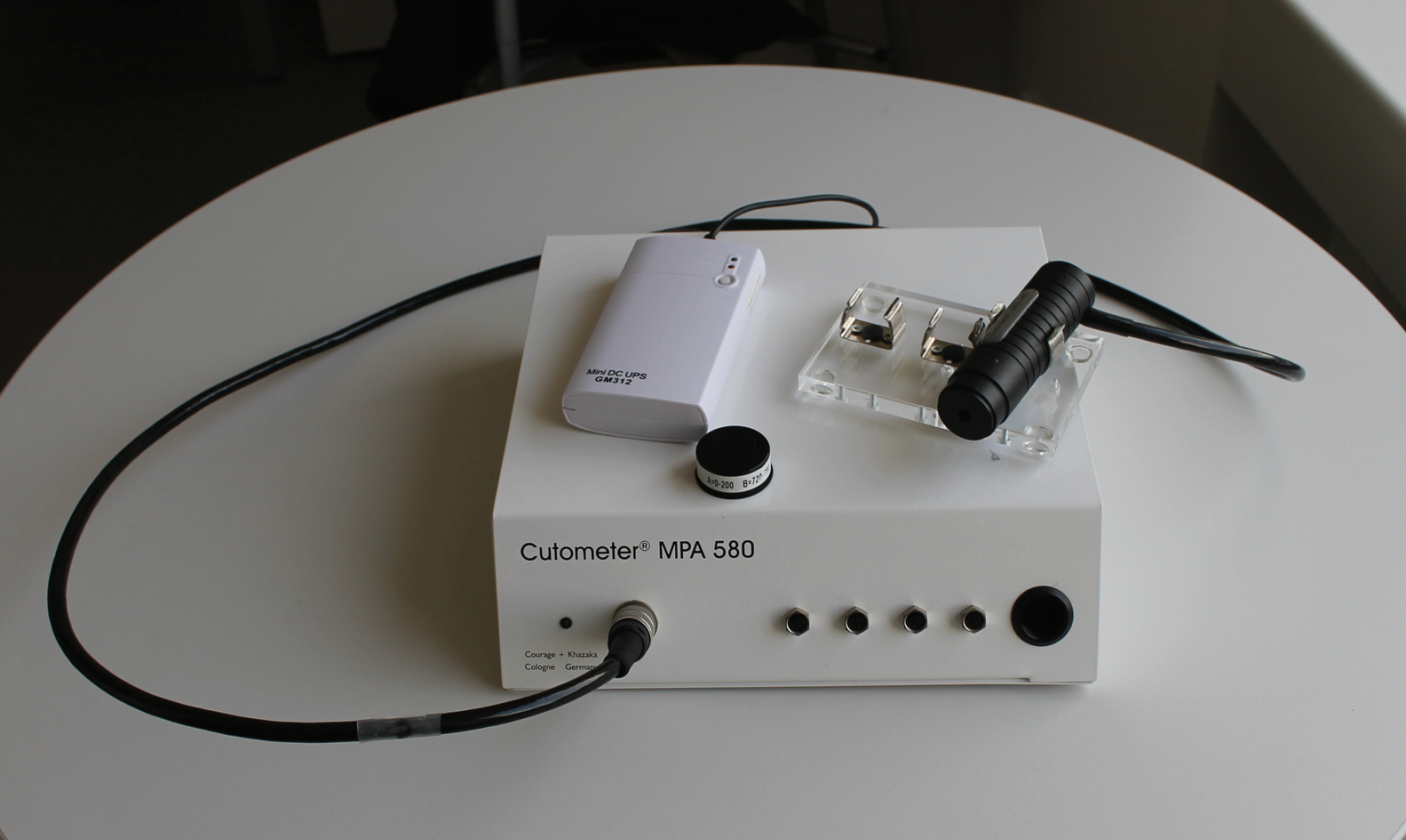
A Cutometer^©^ MPA580 (Courage & Khazaka, Cologne/Germany) including a 6mm probe and customary powerbank for wireless power supply.

**Figure 2.**
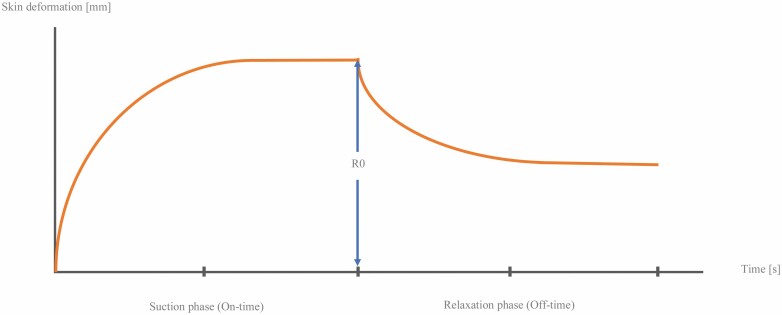
Cutometer output parameter *R*0 representing the maximum skin deformation in mm as result of the applied suction.

The Cutometer^©^ MPA 580 (Courage & Khazaka Electronic GmbH, Cologne, Germany) is a non-invasive suction device developed for the objective and quantitative assessment of mechanical skin properties (Figure 1). It is used for measuring pliability of the affected tissue.^19^ Cutometer^©^ measurements are provided in the form of a curve representing the skin’s response to the applied suction (Figure 2). After application of negative pressure, in the offset period following suction, the deformation of the scar initially does not return to baseline. Therefore, a rest of at least ten minutes between examiners was observed to allow for skin recovery to the original status.

By application of a controlled vacuum for a defined period of time (On-time) using a probe with circular opening of varying diameter (2, 4, 6, 8 mm), the patients skin experiences a vertical deformation, followed by a relaxation period after the negative pressure has been released (Off-time). This process leads to the skin being drawn into the probe aperture during On-time where an infra-red light and corresponding sensor measure the distance to which the skin extends during the suction period and retracts after its release during Off-time. The skin’s response to negative pressure and following release is displayed as elevation-time curves (extension in mm/time) during the measurement by the software Cutometer^©^ Q in real-time. From these curves a number of parameters are calculated representing different quantitative measures of mechanical skin properties as firmness, pliability, and elasticity (Table 1).^20^ Results can be exported to an excel sheet and saved yielding an objective assessment independent of the observer.

**Table 1.** Definition of Cutometer output-parameters

*R*-parameter	Absolute parameter	Definition
*R*0	=Uf	Maximum skin deformation [mm]
*R*1	=Uf Ua	Difference max. deformation/Final retraction
*R*2	=Ua/Uf	Rational retraction/max. deformation
*R*3	=last max. amplitude	If repeated circles of negative pressure/release are applied
*R*4	=last min. amplitude	If repeated circles of negative pressure/release are applied
*R*5	=Ur/Ue	Ration immediate retraction/immediate deformation
*R*6	Uv/Ue	Rationale deformation/immediate deformation
*R*7	Ur/Uf	Ratio immediate retraction/maximum deformation
*R*8	Ua	Final retraction [mm]
*F*0	Area within the curve	Surface parameter
*F*1	Area within the curve	Surface parameter
*Q*0	AUC	Maximum recovery area
*Q*1	*Q*_E_/*Q*_0_	Elastic recovery
*Q*2	*Q*_R_/*Q*_0_	Viscous recovery
*Q*3	(*Q*_E_ + *Q*_R_)/*Q*_0_	Viscoelastic recovery

### Participants

**Figure 3.**
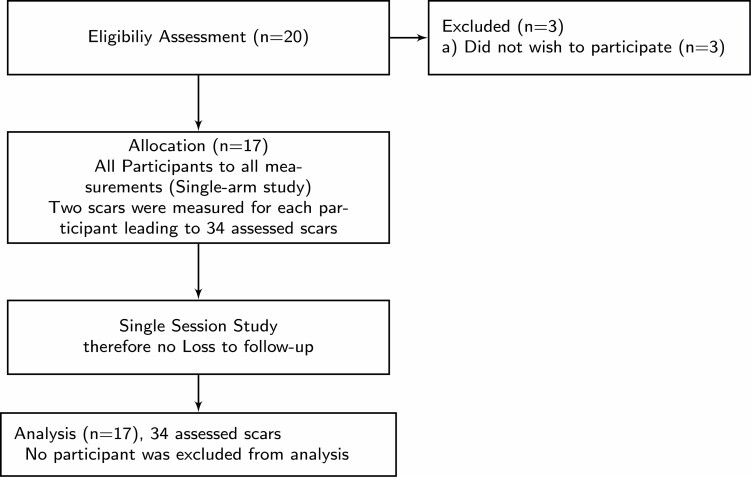
CONSORT diagram of recruitment and analysis.

Patients with burn scars treated at our institution’s burn center were invited to participate in the study if they met the following criteria: 1) age 18 years and above; 2) two or more hypertrophic (yet non-keloid) scar sites of at least 2 months after burn trauma that had been treated conservatively or by grafting of any technique of at least 1 cm^2^ scar size. Patients of any ethnic group were included. Patients were excluded if they suffered from open wounds, pathological skin conditions (psoriasis, eczema, etc.) or psychiatric disorders (ie, dementia or acute psychosis) that would limit their capability for informed consent. Potential participants were identified on multi-disciplinary ward-rounds and through out-patient care. Prior to inclusion, they received detailed information about purpose and design of the study in the form of a patient information leaflet and written informed consent was obtained. Figure 3 shows the recruitment process.

### Measurement Procedures

Four hospital staff members independently assessed two burn scars per patient using the Cutometer^©^ MPA 580 following the standardized measurement protocol developed for this study.

In order to control for the possible influence of topical scar treatments (ointments) on measurements, all scar sites were cleaned with soap prior to the first measurement. To limit the influence of compression or silicone wear, patients were instructed to remove such 30 minutes in advance of the first measurement. For exact relocation of the measurement site, we developed a simple coordinate system comprising two anatomical landmarks for each body region from which the distance to the scar was measured and documented by the first rater. Additionally, two photographs of each scar site were taken: an overview including the anatomical landmarks and a close-up of the measuring site. Both images along with the coordinates were presented to each following rater to enable rapid relocation of the measuring site. In accordance with the literature on burn scars, a Cutometer^©^ probe with a 6-mm aperture and a negative pressure of 450 mbar was applied using the time-strain mode (Mode 1).^9,16–18,21^ The probe applies a vacuum to the skin and measures vertical skin deformation and subsequent retraction in mm/time.

**Figure 4.**
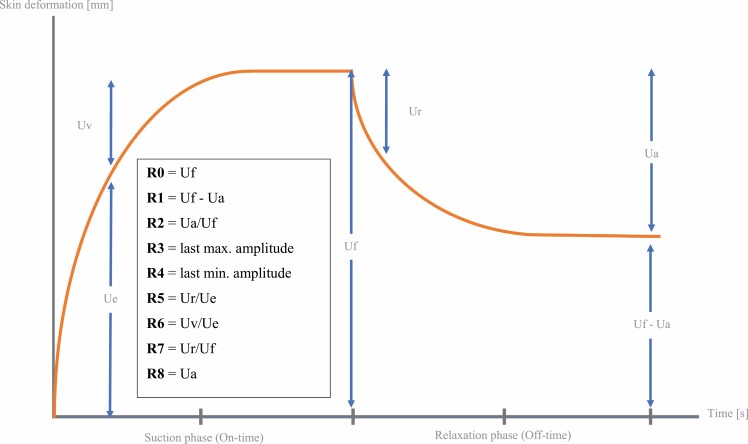
Cutometer output with *R-*parameters representing specific areas of skin recovery.

**Figure 5.**
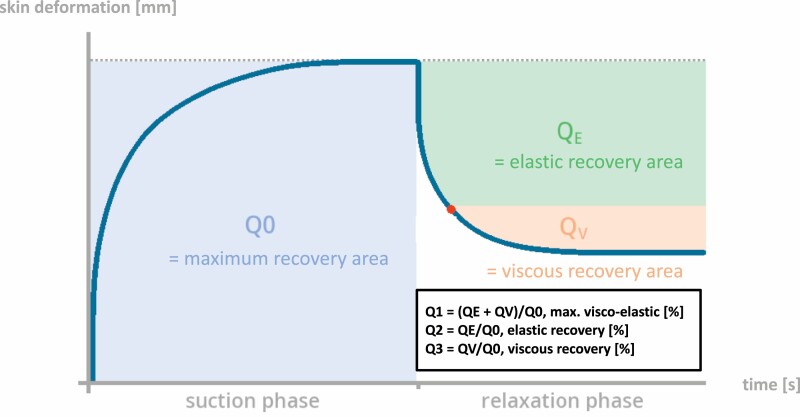
Cutometer output with *Q-*parameters representing specific areas of skin recovery.

**Figure 6.**
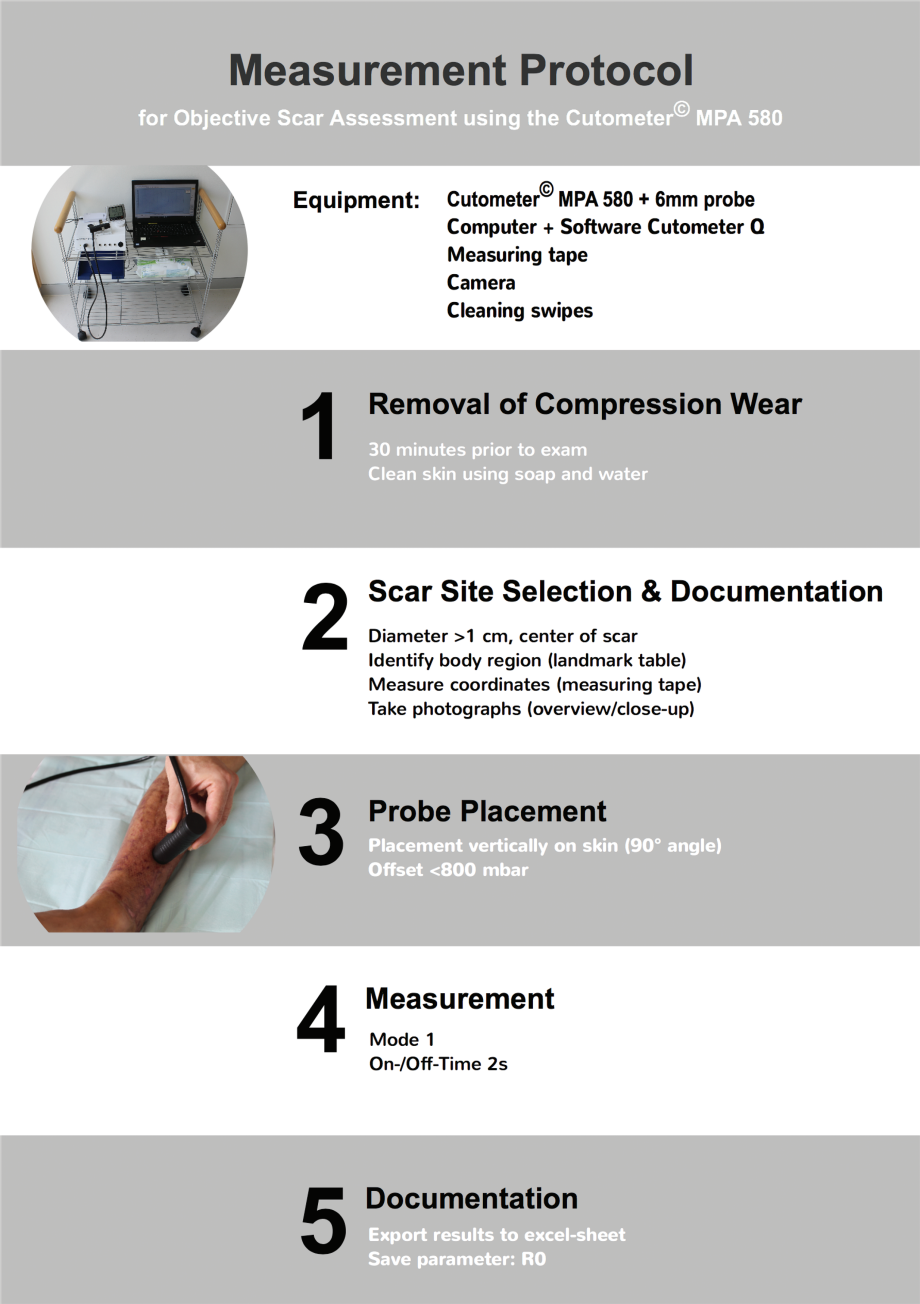
Measurement protocol developed to enable an objective scar assessment at the bedside including documentation of the assessed scar site to enable an exact relocation by consecutive raters. Cutometer (*ICC*, intra-class correlation coefficient).

The skin’s response to this negative pressure and following release is displayed as curves as shown in Figure 4. From these curves, output parameters (Figure 2 and 5) are calculated representing quantitative metrics of mechanical skin properties including firmness, pliability, and elasticity. The force exerted on the measuring probe by the observer or “Offset” has an impact on measurements: moderate-to-heavy additional force has been shown to alternate outcome measures significantly.^21^ Therefore, raters were instructed to apply a maximum probe pressure of 800 mbar following the manufacturer’s recommendation, and Offset was documented after each measurement. Offset in mbar is displayed in real-time for each measurement on the screen by the software Cutometer^©^ Q. Due to this fact ensuring to not exceed the recommended 800 mbar of pressure can be easily controlled by each rater. In some studies, offset-correction was applied calculated by the software Cutometer^©^ Q.^20^ After referring to the manufacturer and carrying out preliminary reliability testing that resulted in higher reliability without the application of Offset-correction, we decided against its application. To evaluate a possible influence of gender on probe pressure, two raters were male (Rater 1 and 3) and two female (Rater 2 and 4). Raters were blinded to previous results. Prior to measurements, room temperature, and relative humidity were measured, and patients were asked to remove compression wear at least 30 minutes prior to the exam. To determine clinical scar severity and demonstrate variability, a VSS-rating for each scar was obtained prior to the first Cutometer^©^ measurement. The final measurement protocol is shown in Figure 6.

### Scar Site Relocation Technique

**Figure 7.**
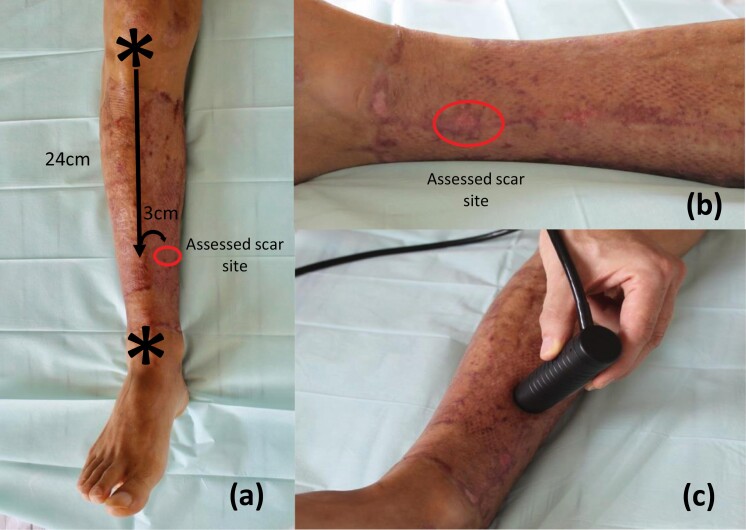
Photographs depicting the determination of *x*/*y*-coordinates based on anatomical landmarks for the lower leg (a) and a close-up for scar site relocation by following raters (b). Afterwards the probe is placed on the skin and after controlling probe pressure (Offset) the measurement is obtained (c).

**Figure 8.**
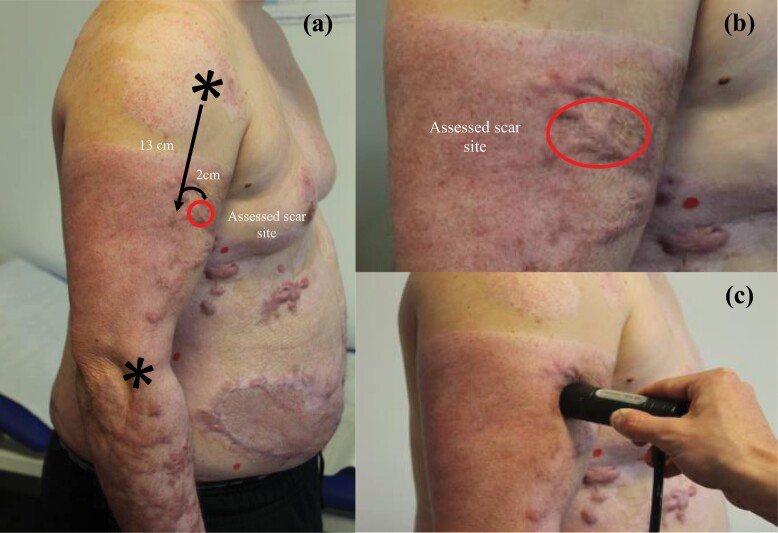
Photographs depicting the determination of *x*/*y*-coordinates based on anatomical landmarks for the upper arm (a) and a close-up for scar site relocation by following raters (b). Afterwards the probe is placed on the skin and after controlling probe pressure (Offset) the measurement is obtained (c).

A major challenge met included finding a reliable yet easy-to-administer method of scar site relocation by consecutive raters to enable a longitudinal assessment over time. In previous studies, relocation procedures were often not reported, not exact or included methods that from our point of view not suited for a longitudinal assessment in clinical practice as using transparent films on which anatomic landmarks were drawn.^17,18,22^ Therefore, we developed a procedure based on defined anatomical landmarks for each body region, an approach familiar to clinicians from orthopedic physical examination. After initial tests had shown that the measured coordinates from landmarks alone were not exact enough as even slight variations caused large differences in Cutometer ratings due to the device’s high sensitivity, we added an overview as well as close-up photograph of the assessed scar site (Figures 7 and 8). After this modification a scar site relocation of consecutive raters with satisfactory precision was achieved. An overview of the applied anatomical landmarks is shown in Table 2.

### Implementation in Clinical Practice

**Figure 9.**
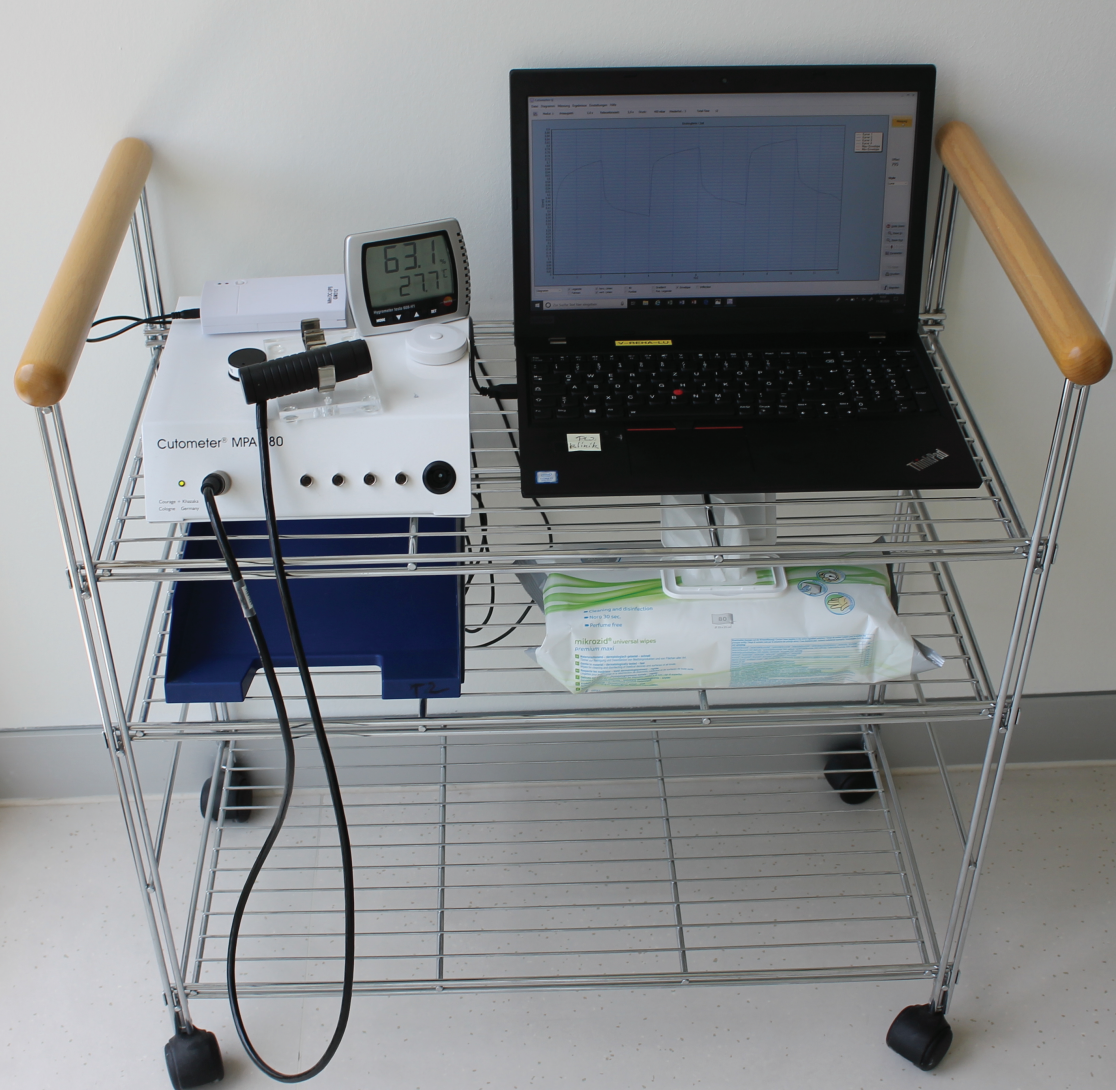
Mobile set-up for bedside scar assessment including a Cutometer^©^ MPA 580 with 6 mm probe and powerbank, a laptop with installed *Cutometer Q* software, measuring tape, thermo- and hygrometer and cleaning swipes.

For application in clinical practice we employed a mobile set-up mounted on a trolley (Figure 9). This is meant to allow time-efficient and easy-to-administer scar assessment at the bedside. The Cutometer^©^ MPA 580 with a 6 mm probe was connected to a customary power bank to provide socket-independent power and connected to a laptop with the installed Cutometer^©^ Q software to document measurement results. Measuring tape and camera for the relocation protocol as well as cleaning swipes and a combined thermometer and hygrometer to assess temperature and humidity prior to the measurement complete the set-up. While getting accustomed to the handling of Cutometer^©^, probe and software prolongs measurements at first, within a couple of completed measurements raters were able to complete an assessment within 15 minutes per scar in addition to the resting period of 30 minutes prior to the exam.

### Outcome

Primary outcome of interest was the validation of our standardized measurement protocol by calculation of an Intra-class Correlation Coefficient (ICC) for all Cutometer^©^ output parameters for average-measure reliability among all raters as well as single-measure reliability. Secondary outcome of interest was to determine the key confounding factors limiting the reliability of Cutometer^©^ measurements on burn scars.

### Statistical Analysis

All statistical analyses were carried out using SPSS Statistics (IBM Corp., Version 26.0. Armonk, NY, USA). All results are expressed as means ± standard deviation (SD) and 95% confidence intervals (CI) where applicable. An alpha level of .05 was chosen for two-sided *P-*values.

To calculate the required sample size, the formula introduced by Bonnet was applied to achieve a sufficient power of .8, at an alpha level of .05 leading to a required minimum of *n* = 31 scars.^23^ To compensate for an assumed drop-out rate of 10% three additional scars were added, leading to an overall number of 34 scars to be assessed. We decided to assess two separate scar areas in each patient leading to a required sample of at least 17 patients. The inter-rater reliability among all four independent raters was determined for each Cutometer output parameter by calculating an ICC using an “absolute agreement” definition in a two-way random effects model.^24–27^ To investigate the influence of rater’s gender on ICC or the applied probe pressure, paired sample *t*-tests were applied.

## RESULTS

### Demographics

A total of 17 participants with burn scars were successfully recruited for the study while three eligible patients (15%) refused participation. In each patient two different scar sites were assessed (*n* = 34). Demographics and scar characteristics of the study population are presented in Tables 3 and 4.

**Table 3.** Demographics of the study population

Characteristic	*n* = 17 patients
Sex	
Male:Female	12:5
Age	
Mean	44.4 years (*SD* = 15.6)
Range	19–66 years
Ethnicity	
Caucasian	13 (82%)
Other	3 (18%)
TBSA	
Mean	36.48% (*SD* = 23.43)
Range	0.7–86.0%

**Table 4.** Scar characteristics, *n* = 34 scars

Characteristic	*n* (%)
Etiology	
Flame	22 (65%)
Scald	10 (29%)
Electrical	2 (6%)
Location	
Head or neck	1 (3%)
Upper arm	6 (18%)
Forearm	5 (15%)
Hand	10 (29%)
Posterior torso	6 (18%)
Anterior torso	2 (6%)
Upper leg	2 (6%)
Foot	2 (6%)
Age of scar (months after burn)	
Median	31.0
Range	3–731
Treatment	
Conservative	4 (13%)
Grafting MESH	24 (71%)
Grafting MEEK	4 (12%)
Full-thickness skin graft	2 (6%)

### Variability of Assessed Burn Scars

A VSS-rating was carried out for each scar.^4^ This rating was not performed as a gold standard, as we believe the limited inter-rater reliability of subjective scar rating scales has been sufficiently established,^8–10^ but solely to demonstrate variability in scar severity among the evaluated scars. The overall median sum score of the individual subscales for vascularity, pigmentation, pliability, and height was 6 (range 3–11) indicating inclusion of low severity scars as well as very severe hypertrophic scars (Table 5).

**Table 5.** Analysis of VSS subscales *n* = 34 scars, *IQR*, interquartile range

Item	Median	IQR	Range
Vascularity	1	[1.00–1.25]	[0–3]
Pigmentation	2	[1.00–2.00]	[0–2]
Pliability	2.5	[1.75–3.00]	[1–4]
Height	1	[1.00–2.00]	[0–3]
Score	6	[5.00–8.00]	[3–11]

### Objective Scar Assessment

**Figure 10.**
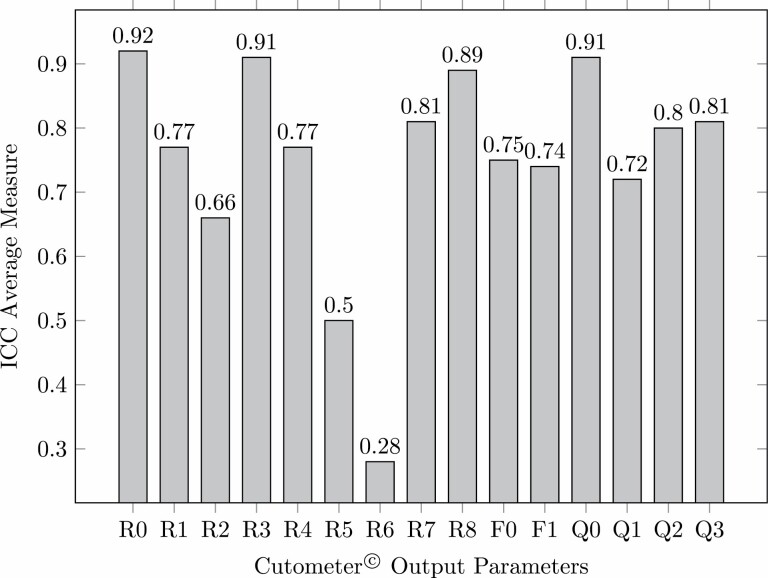
Inter-rater reliability for Cutometer output parameters for average among raters (Average-Measure), ICC, intra-class correlation coefficient. ICC-values > 0.90 indicate excellent, > 0.80 good, < 0.70 acceptable and < 0,70 below acceptable reliability.

ICC values and their 95% confidence interval were calculated based on an average rating of all raters “Average Measure” as well as individual rating “Single Measure” of *k* = 4 raters in an Absolute-agreement, Two-Way Random model. The results for Average-Measure ICC are presented in Table 6 and Figure 10. The statistical analysis showed excellent inter-rater reliability (> .90) for the three output parameters *R*0 (.918), *R*3 (.910) and *Q*0 (.914). Good inter-rater reliability (> .80) was demonstrated for parameters *R*7 (.815), *R*8 (.899), and *Q*2 (.803) as well as *Q*3 (.806). For parameters *R*1 (.767), *R*4 (.767), *F*0 (.749), *F*1 (.737) and *Q*1 (.720) and acceptable inter-rater reliability (> .70) was obtained. All other output parameters fell below the threshold of acceptable inter-rater reliability.

**Table 6.** ICC Calculation for *R*-parameters for Average-Measure *n* = 34 scars

*R*-parameter	ICC (2,1) Average-Measure	95% CI	*P*
*R*0	0.918	[0.862; 0.956]	< .001
*R*1	0.767	[0.606; 0.873]	< .001
*R*2	0.663	[0.430; 0.816]	< .001
*R*3	0.910	[0.847; 0.951]	< .001
*R*4	0.767	[0.606; 0.873]	< .001
*R*5	0.495	[0.142; 0.725]	.01
*R*6	0.284	[−0.210; 0.609]	.11
*R*7	0.815	[0.687; 0.899]	< .001
*R*8	0.899	[0.829; 0.945]	< .001
*F*0	0.749	[0.577; 0.863]	< .001
*F*1	0.737	[0.559; 0.856]	< .001
*Q*0	0.914	[0.853; 0.953]	< .001
*Q*1	0.720	[0.526; 0.847]	< .001
*Q*2	0.803	[0.667; 0.892]	< .001
*Q*3	0.806	[0.674; 0.894]	< .001

**Figure 11.**
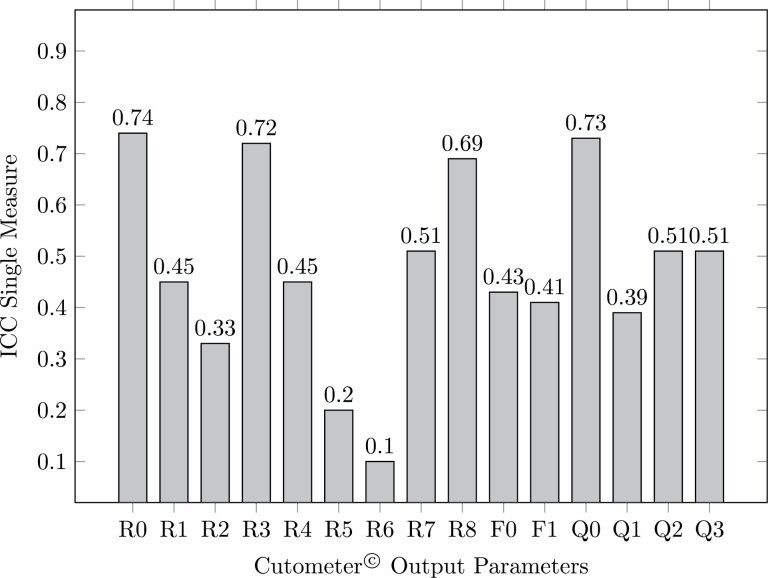
Inter-rater reliability for Cutometer output parameters for individual rater reliability (Single-Measure), ICC, intra-class correlation coefficient. ICC-values > 0.90 indicate excellent, > 0.80 good, < 0.70 acceptable and < 0.70 below acceptable reliability.

The inter-rater reliability for an individual rater is shown in Table 7 and Figure 11. The calculated ICC values showed a lower inter-rater reliability as compared to Average-Measure results. However, even for Single-Measure analysis, three output parameters showed acceptable inter-rater reliability (> .70). The highest inter-rater reliability was obtained for parameter *R*0 (.738), followed by parameter *Q*0 (.726) and *R*3 (.715). Temperature [°C] and humidity [%] were documented before each scar assessment. Average temperature was 23.4 ± 1.18°C ranging from 21.9 to 26.9°C. Mean humidity was 36.8 ± 7.6% ranging from 24.0 to 51.3%.

**Table 7.** ICC calculation for *R*-parameters for Single-Measure *n* = 34 scars

*R*-parameter	ICC (2,1) Single-Measure	95% CI	*P*
*R*0	0.738	[0.609; 0.843]	< .001
*R*1	0.452	[0.278; 0.632]	< .001
*R*2	0.330	[0.159; 0.527]	< .001
*R*3	0.715	[0.581; 0.828]	< .001
*R*4	0.452	[0.277; 0.632]	< .001
*R*5	0.197	[0.040; 0.397]	.01
*R*6	0.090	[−0.045; 0.280]	.11
*R*7	0.505	[0.326; 0.683]	< .001
*R*8	0.691	[0.549; 0.812]	< .001
*F*0	0.427	[0.254; 0.611]	< .001
*F*1	0.412	[0.240; 0.597]	< .001
*Q*0	0.726	[0.591; 0.836]	< .001
*Q*1	0.391	[0.217; 0.581]	< .001
*Q*2	0.505	[0.333; 0.675]	< .001
*Q*3	0.510	[0.341; 0.678]	< .001

### Offset

Since the force exerted on the measuring probe by the observer (Offset) has a significant impact on measurements,^21^ raters were instructed to apply a maximum probe pressure of 800 mbar following the manufacturer’s recommendation. Offset was documented after each measurement. Mean probe pressure was 367.4 ± 166.1 mbar with a range from 173 to 1053 mbar. In three cases the manufacturer-recommended probe pressure limit of 800 mbar was exceeded. After excluding these cases from analysis, the calculated inter-rater reliability (ICC) increased further as shown in Table 8.

**Table 8.** ICC results after exclusion of measurements with probe pressure exceeding 800 mbar *n* = 31 scars

Parameter	ICC (2,1) “Average Measure”	ICC (2,1) “Single Measure”	*P*
*R*0	0.932 [0.883; 0.964]	0.775 [0.654; 0.871]	< .001
*R*3	0.925 [0.869; 0.960]	0.755 [0.624; 0.858]	< .001
*Q*0	0.938 [0.892; 0.967]	0.791 [0.673; 0.881]	< .001

### Influence of Gender

In order to determine whether gender has an influence on inter-rater reliability of Cutometer^©^ ratings or the applied probe pressure, paired sample *t*-tests comparing the average results from female and male raters (*n* = 2 each) were conducted. Following an average-measure definition for the evaluation of all fifteen Cutometer^©^ output parameters, there was no significant difference in means of ICC-values for male (0.681 ± 0.131) and female (0.634 ± 0.216) raters; *t*(14) = 0.888, *P* = .390, 95% CI [−0.068; 0.163]. Also, for ICC-values following a single-measure definition no significant difference in ICC-values for male (0.531 ± 0.170) and female (0.493 ± 0.202) raters was detected; *t*(14) = 0.760, *P* = .460, 95% CI [−0.069; 0.145]. In addition, for probe pressure no significant difference between Offset-values for male (361.04 ± 137.20 mbar) or female (373.81 ± 139.60 mbar) raters could be detected; *t*(33) = 0.733, *P* = .469, 95% CI [−22.666; 48.195]. In conclusion, gender did not have a significant influence on probe pressure or inter-rater reliability of measurements in our study.

## DISCUSSION

Severe burn scars can lead to lifelong sequelae including pain, disability, and mental strain.^1^ Despite these potentially devastating consequences, the clinical management of burn scars still relies on subjective scar assessment scales with often poor reliability.^7,9,11,22^

Reliability among different examiners becomes particularly relevant for scar monitoring tools used in clinical out-patient care, as over time, each individual scar is usually assessed by multiple clinicians. To confidently draw conclusions from those assessments, tools are required to provide sensitive and user-independent metrics for relevant scar tissue characteristics. Although modern skin assessment devices, regularly used for research purposes, fulfill these requirements, their clinical applicability is limited by elaborate and time-consuming measurement protocols. To address this issue, we introduced and validated a brief, standardized measurement procedure for the Cutometer, to provide a quantitative assessment of burn scars, readily applicable in clinical practice.

In general, we found the Average-Measure Intra-class correlation coefficients (ICC) for the Cutometer output parameters to be significantly higher than those calculated for Single-Measure. These findings are not surprising, since an Average-Measure ICC represents the reliability among the averaged measures of multiple raters (in our study *k* = 4) that will always be higher than mean reliability for an individual rater, which is represented by Single-Measure ICC.

For Average-Measure, three parameters obtained excellent reliability (*R*0, *R*3, and *Q*0), four good (*R*7, *R*8, *Q*2, and *Q*3) and an additional five parameters showed acceptable reliability (*R*1, *R*4, *F*0, and *F*1). In contrast, for Single-Measure analysis only three parameters (*R*0, *R*3, and *Q*0) were of acceptable inter-rater reliability. However, with regard to employment in a clinical setting the values for Single-Measure are of higher relevance as in everyday clinical practice usually only a single clinician is available to assess a scar at a time. Therefore, only output parameters that would obtain at least acceptable inter-rater reliability in a Single-Measure ICC analysis should be considered for application in a clinical setting. By achieving acceptable inter-rater reliability for the three parameters *R*0, *R*3, and *Q*0 we demonstrated the superiority of Cutometer-based objective scar assessment as compared to subjective scar rating scales as VSS or POSAS that always fell below the threshold of acceptable reliability (ICC > 0.70) in previous studies.^9–11,22^

Consequently, the parameters that should be considered are *R*0 as an indicator of pliability or firmness, *R*3 representing the last maximum amplitude of the output curve and *Q*0. Among those we particularly recommend the parameter *R*0 as it showed the highest inter-rater reliability of all fifteen parameters calculated by the Cutometer^©^ software. Furthermore, *R*0 is the most frequently recommended output parameter in the literature on Cutometer^©^ scar assessment.^7,12,17^ Moreover, the scar’s firmness or pliability, which it represents, is of particular clinical relevance as it directly corresponds to functional range of motion essential for daily living skills as well as return to the workplace.

Parameter *R*3 should not necessarily be considered for scar assessment as it showed the lowest inter-rater reliability of the three and offers little additional information to *R*0. Moreover, as an indicator of fatigue effects of the skin following repeated suction, it is of technical rather than clinical interest. If additional information on elasticity of the affected tissue is required, output parameter *Q*0 could be considered, that has, to the best of our knowledge, not been tested previously with regard to scar assessment. As a secondary outcome, we investigated the influence of the rater’s gender on the reliability of Cutometer ratings. We found no difference in reliability between male and female raters. As a limitation, since two scar sites in each patient were assessed, a possible influence on the applied t-test due to this dependency cannot be excluded.

The applied probe pressure, or “Offset”, is known to have a significant influence on the measured skin properties. Thus, we decided to exclude those measurements were the probe pressure exceeded 800 mbar. Comparison of the ICC before and after exclusion revealed significant differences between groups indicating that excessive probe pressure, above the manufacturer-recommended 800 mbar, affects measurement reliability. We therefore recommend controlling for probe pressure below 800 mbar for burn scar assessment. Further, mechanical skin properties, in particular those of the epidermis are influenced by environmental conditions such as ambient temperature and humidity.^21^ Both metrics are difficult to control in a clinical setting. Thus, we aimed to prove the reliability among a typical real-world ambient temperature and humidity range in clinical practice (22–27°C and 24–51% relative humidity). Even though the study design did not allow for detailed analysis of the influence of temperature and humidity, we found that not controlling temperature and humidity did not prevent reliable measurements using the Cutometer^©^ MPA 580 in our study.

The applied algorithm has been proven to provide consistency among scars of varying severity, indicating its broad applicability. With regard to the study design, the assumed drop-out rate of 10% was rather small. However, due to the close patient monitoring and guidance in our burn center, this rather modest drop-out rate was assumed. As a limitation, this study did not include serial scar assessments over time. However, to determine the inter-rater reliability of diagnostic tests, a single-measure design provides conclusive results with reasonable time- and cost-effectiveness and was therefore our first choice. In a next step, we evaluate the algorithm for serial scar assessments as part of an ongoing longitudinal study in the study center.^28^

In conclusion, following the introduced standardized measurement protocol for the Cutometer^©^ MPA 580 an objective burn scar assessment with acceptable reliability for a single rater and excellent reliability for multiple raters can be obtained. In agreement with the literature, our findings indicate the use of the output parameter *R*0 as a metric for scar firmness and pliability due to its high reliability and clinical relevance to the functional assessment of a scar. If additional information on the scar’s elasticity is required, the parameter *Q*0 can be considered as well. Probe pressure (“Offset”) should not exceed 800mbar. Rater gender does not seem to have a significant influence on probe pressure or inter-rater reliability. Further studies are required to investigate if our findings apply to the longitudinal assessment of scars and non-burn scars as well. In conclusion, for the first time a validated protocol for an objective and reliable scar assessment focused on applicability in clinical practice is presented in this study.

**Table 2.** Overview of anatomical landmarks for scar site relocation

Body region	Patient’s position	First anatomical landmark	Second anatomical landmark
Trunk ventral	Lying on the back, head straight	Jugular notch	Pubic symphysis
Trunk dorsal	Standing, arms hanging down sideways	Spinous process	C7 Anal cleft
Upper arm	Standing, arms hanging down sideways	Acromion	Humerus, lateral epicondyle
Lower arm	Standing, arms hanging down sideways	Olecranon	Ulna, styloid process
Hand	Palm lying on flat, fingers closed	Lister’s tubercle	D3, head
Upper leg	Lying on the back, legs straight	Anterior superior iliac spine	Patella, upper end
Lower leg	Lying on the back, legs straight	Patella, lower end	Median medial/lateral malleolus
Foot	Planted on the floor	Median medial/lateral malleolus	D3, distal phalanx
Head	Sitting upright, head straight	Spinous process C7	Tip of the nose
Face	Lying on the back, head straight	Tip of the nose	Mental protuberance
Neck ventral	Lying on the back, head straight	Mental protuberance	Jugular notch
Neck dorsal	Standing, arms hanging down sideways	Occipital protuberance	Spinous process C7

## Notes

Ethics Declaration: This study was approved by the competent ethics committee of Rhineland-Palatinate, Germany, reference number 837.236.16 (10555). Written, informed consent from all participants in the study was obtained prior to participation.

Funding: Leila Harhaus received a research grant from the German Statutory Accident Insurance (DGUV, Deutsche Gesetzliche Unfallversicherung) for the study “Evaluation eines ICF-orientierten Rehabilitationskonzepts thermische Verletzungen” (Evaluation of an ICF-based burn rehabilitation concept for thermal injuries) of which this study is sub-study. None of the authors has a financial interest in any of the products, devices, or drugs mentioned in this manuscript.
